# *Ureaplasma parvum* Septic Arthritis, a Clinic Challenge

**DOI:** 10.3390/diagnostics12102416

**Published:** 2022-10-06

**Authors:** Carlos Suárez-Cuervo, Concepción Nicolás, Jonathan Fernández-Suárez, Ana Morilla, Javier Fernández, Luis Caminal-Montero

**Affiliations:** 1 Department of Internal Medicine, Hospital Universitario de Cruces, 48903 Bilbao, Spain; 2 Department of Haematology, Hospital Universitario Central de Asturias, 33011 Oviedo, Spain; 3 Department of Clinical Microbiology, Hospital Universitario Central de Asturias, 33011 Oviedo, Spain; 4 Traslational Microbiology Group, Instituto de Investigación Sanitaria del Principado de Asturias (ISPA), 33011 Oviedo, Spain; 5 Research & Innovation, Artificial Intelligence and Statistical Department, Pragmatech AI Solutions, 33001 Oviedo, Spain; 6 Centro de Investigación Biomédica en Red-Enfermedades Respiratorias, 28220 Madrid, Spain; 7 Department of Internal Medicine, Hospital Universitario Central de Asturias, 33011 Oviedo, Spain; 8 Group of Basic and Translational Research in Inflammatory Diseases, Instituto de Investigación Sanitaria del Principado de Asturias (ISPA), 33011 Oviedo, Spain

**Keywords:** *Ureaplasma parvum*, septic arthritis, culture-negative, doxycycline

## Abstract

*Ureaplasma parvum* is usually part of the normal genital microbiota. Rarely, it can cause invasive infections such as septic arthritis or meningitis. A case of a 74-year-old woman with follicular lymphoma who developed cellulitis followed by elbow arthritis with negative routine bacterial cultures is described. *U. parvum* was identified in the synovial fluid using a broad-range 16S ribosomal RNA gene polymerase chain reaction (PCR) and also in vaginal fluid by a targeted PCR (Anyplex™ II STI-7). Multilocus Sequence Typing (MLST) revealed that isolates from both sources belonged to ST4, a worldwide distributed clone. Treatment consisted of surgery and targeted antibiotic therapy with doxycycline and azithromycin. Evolution showed initial clinical improvement in arthritis despite functional sequelae. *Ureaplasma* arthritis should be considered as a rare cause of arthritis in negative culture, especially in immunosuppressed patients. In these cases, the treatment is not well established, but according to this and previous works, patients could improve with doxycycline, azithromycin or fluoroquinolone therapy on a prolonged basis.

## 1. Introduction

*Ureaplasma parvum*, previously known as *Ureaplasma urealyticum* biovar 1, are small, organisms bacteria belonging to the class *Mollicutes*, which commonly colonize the urogenital tract in humans and have well-established pathogenicity in urogenital infections [1]. Their ability to cause disease in hosts with competent immune status remains unclear. It has not been until recently that *Ureaplasma* spp. has been reported as an uncommon etiological agent responsible for arthritis, especially in prosthetic joint infections and immunocompromised hosts [2]. Due to their microbial features and growth requirements, these bacteria are not detected by routine cultures in clinical microbiology laboratories [1]. They are resistant to several antibiotics including those commonly prescribed for septic arthritis. Due to the fact that they lack peptidoglycan, they are resistant to vancomycin, β-lactams and other drugs that act on the bacterial wall synthesis [3]. In addition, they are resistant to sulfonamides and trimethoprim because they do not synthesize folic acid.

The aim of this work is to describe a new case of *U. parvum* septic arthritis affecting a 74-year-old immunosuppressed woman with cirrhosis, diagnosed and treated for lymphoma, and to perform molecular typing of the causal agent.

## 2. Case

A 74-year-old woman with cryptogenic liver cirrhosis was admitted to the Hospital Universitario Central de Asturias (HUCA) in July 2020, due to progression of lymphoma in July 2020. She had received treatment for follicular lymphoma with R-CHOP from October 2013 to April 2016. She was doing well until two months before presentation, after developing peripheral edema. A body computerized tomography scan showed recurrence of the lymphoma, which was confirmed by an inguinal lymphadenopathy biopsy.

Serum analysis showed low serum immunoglobulin values: IgG 1.69 g/L (reference range: 7.67–15.9), IgA 0.54 g/L (reference range: 0.61–3.56), and IgM 0.10 g/L (reference range: 0.37–2.86). Leukocytes count was 600 × 10^3^ µL (lymphocytes 22%). Physical examination revealed mental confusion and fever, ascites, and a warm and erythematous swelling over the right elbow with a limited range of motion, and inflammatory signs in her thighs, with redness, warmth, tenderness, and swelling of the skin, clinically suggestive of cellulitis. A paracentesis was performed and peritonitis was ruled out. She was diagnosed with elbow cellulitis and treated with amoxicillin-clavulanate. In addition, septic arthritis was suspected, and an ultrasound-guided right elbow arthrocentesis was performed. Synovial fluid showed 15.500 white blood cells per mm^3^ with 89% neutrophils. The antibiotic was switched to vancomycin plus piperacillin-tazobactam as broad-spectrum empiric therapy for septic arthritis. Microscopic examination of joint aspirate was negative for crystals and the Gram-stain-negative for bacteria. Blood cultures were also negative, as well as bacterial, mycobacterial, and fungal cultures and the FilmArray^®^ Blood Culture Identification Panel (FA BCID) (BioFire/bioMérieux, Salt Lake City, UT, USA) directly applied to synovial fluid. However, a broad-range 16S ribosomal RNA gene PCR and further sequencing performed directly from sample, as previously described [4], identified the presence of *U. parvum* (GenBank accession number OP432080). Patient denied genital complaints, and the gynecologic examination was normal. After demonstration of septic arthritis, the multiplex PCR Anyplex™ II STI-7 (Seegene, Seoul, Korea) for diagnosis of sexually transmitted infections was performed both in the vaginal and the synovial fluid, being positive for *U. parvum* in both samples.

Samples were inoculated in Urea-Arginine LYO 2 broth (Biomérieux, Marcy l’Étoile, France) and incubated 24 h at 37 °C. Bacterial DNA was extracted from the inoculated culture broth using a MagCore extractor (RBC Bioscience, New Taipei City, Taiwan), and expanded Multilocus Sequence Typing (eMLST) was performed for epidemiological investigation. PCR was accomplished using primers and conditions previously described [5], amplicons were purified using ExoSAP-IT (USB Corporation, Cleveland, OH, USA), and the two DNA strands were sequenced (Eurofins Genomics, Ebersberg, Germany). Both isolates belonged to the ST4 clone.

Elbow magnetic resonance imaging revealed a fluid collection, synovial proliferation, and cellulitis without osteomyelitis. The patient underwent an elbow arthroscopy with washed out, debridement, and evacuation of grossly purulent fluid, persisting with fever. She started azithromycin plus doxycycline therapy for two weeks. Azithromycin was subsequently discontinued, whereas she received doxycycline for another four weeks. Since the introduction of targeted treatment against *Ureaplasma*, the patient became afebrile with progressive improvement in inflammatory signs in her elbow. She was discharged in October, but despite treatment with rituximab and immunoglobulins, the evolution of her basic condition was unfavorable, suffering further infections and dying three months later.

## 3. Discussion

The *Mycoplasmataceae* family comprises two genera, *Mycoplasma* and *Ureaplasma*. The latter can cause respiratory or urogenital infections and self-limited postpartum fever, but infections in adults outside the urogenital tracts are less frequent and may be unrecognized [6]. These organisms are difficult to isolate and require special culture media and conditions. PCR targeting *Ureaplasma* spp. is the preferred diagnostic method because of its high sensitivity and rapid turnaround and is routinely performed in urogenital specimens in several clinical microbiology laboratories [7,8]. *U. parvum* is considered part of the normal genital microbiota due the high prevalence of colonization in the genital tract of a healthy female, which is up to 40% [9]. Thus, it is usually not reported in genital samples because of its controversial clinical significance. Conversely, *U. urealyticum* is considered more pathogenic being a known agent of non-gonococcal urethritis and perinatal infections [1].

Mycoplasmas in general, including *Ureaplasma* spp. and *Mycoplasma* spp., have been reported as an uncommon but important cause of septic arthritis, especially affecting immunosuppressed patients [10,11]. Risk factors for this condition include immunosuppression, prosthetic joints, hematologic malignancies, organ transplant, and urogenital manipulation. Apart from the present report, only nine cases of septic arthritis due to *U. parvum* have been described in the last few years [2,12,13,14,15,16,17,18,19]. Main clinical features of all of them are summarized in Table 1. The 83% of the affected patients were males, and all the cases have been reported as part of systemic infections. The first one, reported in 2010, was a bilateral knee arthritis with aortic co-infection by *U. parvum* and *M. hominis* in a man with lymphoma, who also had mycotic aneurysms [16]. Four out of nine reported cases occurred in immunocompromised patients, and only two affected patients with prosthetic joints. The joints involved were the knees, shoulders, and hips. The mechanism or route of joint infections by mycoplasmas is unknown. Unlike other studies, the presence of this microorganism in the patient’s vagina was demonstrated, and both isolates belonged to the same clone (ST4). Thus, the most plausible mechanism of infection in case reported herein could be vaginal colonization followed by bacteremia and further migration to the elbow favored by the patient’s immune weakness. The clonality study gives more strength to the hypothesis that the commensal strains are the ones that subsequently infect the joints. There are only a few works where the clonality of *Ureaplasma* spp. has been analyzed, and in several of those studies, ST4 was found among one of the most frequent clones of *U. parvum* in the genital tract of women, being one of the most prevalent clones in China, Switzerland, and the United States [3,5,20].

Most *Mycoplasma* and *Ureaplasma* infections are treated empirically. The optimal treatment of *U. parvum* septic arthritis is poorly defined [2,21]. Three out of the nine patients with *U. parvum* joint infections previously reported were successfully treated with doxycycline, while the remaining four received moxifloxacin or macrolides. Fluoroquinolones, tetracyclines, and macrolides are the most effective drugs against *Ureaplasma* spp. [1]. Furthermore, dual treatment using tetracyclines, usually doxycycline, and a macrolide or levofloxacin may be an option in selected cases. The association of a fluoquinolone was ruled out in the present case since several works have documented fluoroquinolone-resistant isolates of *U. parvum* in different parts of Europe, America and Asia [3,21,22].

Recommendations for the duration of treatment of *Mycoplasma* and *Ureaplasma* septic arthritis is not well established but ranged from weeks to months in the reviewed literature.

## 4. Conclusions

Mycoplasmas, and specifically *U. parvum*, are an uncommon cause of septic arthritis but should be screened in immunosuppressed patients and prosthetic infections, especially in those cases with negative bacterial routine cultures. Broad-range 16S rRNA gene sequencing or targeted PCR are essential tools for the diagnosis of this kind of culture-negative infections. Although there is little evidence to guide their management, long regimens with doxycycline, macrolides, or fluoroquinolones have been used successfully to treat these infections.

## Figures and Tables

**Table 1:** Clinical features of patients with septic arthritis caused by *Ureplasma parvum*.

Age/ Sex	Comorbidities/Risk Factors	Joint Involved	Possible Transmission and/or Source of Infection	Final Therapy	Reference/ Year
74/F	Lymphoma	Elbow	Vaginal infection/colonization	Doxyccyline	Current study
57/F	Unknown	Hip, Knee	Unknown	Moxifloxacin	[19]/2022
72/M	Unknown	Knee	Unknown	Doxyccyline	[18]/2021
39/M	Rituximab therapy	Elbow	Bladder fistula and prostatic abscess	Moxifloxacin	[17]/2021
56/F	Lymphoma therapy	Knee	Ureterocystitis	Clarithromycin	[13]/2021
68/F	Unknown	Knee	Joint protein-rich plasma injection	Azithromycin	[12]/2020
65/M	Unknown	Knee	Unknown	Doxyccyline	[2]/2019
56/M	Lymphoma therapy	Shoulder	Orchitis	Doxyccyline	[15]/2017
75/M	Unknown	Knee	Unknown	Doxyccyline	[14]/2014
54/M	Hypogammabloblulinaemia	Hip, prosthetic knee	Unknown	Moxifloxacin	[16]/2010

F, female; M, male.

## Data Availability

Ureaplasma 16S DNA sequence has been deposited in GenBank (accession number OP432080).
